# Association between cell-bound blood amyloid-β(1–40) levels and hippocampus volume

**DOI:** 10.1186/s13195-014-0056-3

**Published:** 2014-10-20

**Authors:** Oscar Sotolongo-Grau, Pedro Pesini, Sergi Valero, Asunción Lafuente, Mar Buendía, Virginia Pérez-Grijalba, Itziar San José, Marta Ibarria, Miguel A Tejero, Joan Giménez, Isabel Hernández, Lluís Tárraga, Agustín Ruiz, Mercé Boada, Manuel Sarasa

**Affiliations:** 1 Alzheimer Research Center and Memory Clinic, Fundació ACE Memory Clinic, Institut Català de Neurociències Aplicades, Marquès de Sentmenat, 57, Barcelona, 08029, Spain; 2 Araclon Biotech Ltd, Via Hispanidad 21, Zaragoza, 50009, Spain; 3 Department of Psychiatry, Hospital Universitari Vall d’Hebron, Universitat Autònoma de Barcelona, Passeig Vall d’Hebron, 119–129, Barcelona, 08035, Spain; 4 Department of Diagnostic Imaging, Clínica Corachan, Buïgas, 19, Barcelona, 08017, Spain

## Abstract

**Introduction:**

The identification of early, preferably presymptomatic, biomarkers and true etiologic factors for Alzheimer’s disease (AD) is the first step toward establishing effective primary and secondary prevention programs. Consequently, the search for a relatively inexpensive and harmless biomarker for AD continues. Despite intensive research worldwide, to date there is no definitive plasma or blood biomarker indicating high or low risk of conversion to AD.

**Methods:**

Magnetic resonance imaging and β-amyloid (Aβ) levels in three blood compartments (diluted in plasma, undiluted in plasma and cell-bound) were measured in 96 subjects (33 with mild cognitive impairment, 14 with AD and 49 healthy controls). Pearson correlations were completed between 113 regions of interest (ROIs) (45 subcortical and 68 cortical) and Aβ levels. Pearson correlation analyses adjusted for the covariates age, sex, apolipoprotein E (ApoE), education and creatinine levels showed neuroimaging ROIs were associated with Aβ levels. Two statistical methods were applied to study the major relationships identified: (1) Pearson correlation with phenotype added as a covariate and (2) a meta-analysis stratified by phenotype. Neuroimaging data and plasma Aβ measurements were taken from 630 Alzheimer’s Disease Neuroimaging Initiative (ADNI) subjects to be compared with our results.

**Results:**

The left hippocampus was the brain region most correlated with Aβ(1–40) bound to blood cell pellets (partial correlation (pcor) = −0.37, *P* = 0.0007) after adjustment for the covariates age, gender and education, ApoE and creatinine levels. The correlation remained almost the same (pcor = −0.35, *P* = 0.002) if phenotype is also added as a covariate. The association between both measurements was independent of cognitive status. The left hemisphere entorhinal cortex also correlated with Aβ(1–40) cell-bound fraction. AB128 and ADNI plasma Aβ measurements were not related to any brain morphometric measurement.

**Conclusions:**

Association of cell-bound Aβ(1–40) in blood with left hippocampal volume was much stronger than previously observed in Aβ plasma fractions. If confirmed, this observation will require careful interpretation and must be taken into account for blood amyloid-based biomarker development.

## 1 Introduction

Alzheimer’s disease (AD) is the most frequent cause of dementia in Western societies. Neuropsychological evaluation remains the most useful tool for the diagnosis of AD and mild cognitive impairment (MCI) [1]. However, new biomarkers, such as amyloid-β protein fragment 1–42 (Aβ42) and phospho-tau protein levels in cerebrospinal fluid (CSF) measured by magnetic resonance imaging (MRI)–based hippocampal volumetry, have also been proposed [2]. More recently, direction of Aβ or tau using radiotracers in positron emission tomography has emerged as a promising candidate for improving diagnosis and monitoring drug treatments and disease progression.

Although Aβ levels in plasma have been widely investigated as a potential biomarker for AD evaluation, the development of a blood-based test to diagnose AD has remained elusive. Therefore, no definitive plasma or blood biomarker that can be used to indicate high or low risk of conversion to AD has been confirmed to date [3]. Importantly, Aβ peptides in blood can be found free in the plasma, bound to plasma proteins and bound to blood cells [4]. There are several enzyme-linked immunosorbent assay (ELISA)–based tests to measure only free Aβ in plasma [5]-[8]. In fact, most studies reported to date have been related to the measurement of Aβ levels in plasma fraction [9]-[11]. However, as the majority of Aβ peptides are bound to blood cells [12], a comprehensive Aβ blood test must include the determination of peptide levels in each of these three fractions.

Our group is actively involved in the development of novel sandwich ELISA colorimetric tests for detection of Aβ using whole blood instead of plasma alone [4],[13]. In fact, this technology was used in a recently conducted trial, titled the AB128 project, in which we studied Aβ blood level as a potential AD biomarker. Specifically, we found statistically significant differences in some measurements in different blood compartments when we data from compared healthy controls (HCs) and subjects with MCI [14].

In this work, we aim to establish a relationship between blood Aβ levels obtained using these novel ELISA techniques and brain morphometry measured using MRI in healthy and cognitively impaired individuals. We postulated that if the blood Aβ load is associated with AD, those biomarker levels could be related to brain regions of interest (ROIs) previously defined for AD. Therefore we undertook a data exploration to identify blood Aβ measurement methods which correlated with brain volume.

## 2 Methods

### 2.1 Study population

The study included 96 participants divided into three clinical groups based on their cognitive status, comprising 33 patients with amnesic MCI, 14 patients with AD and 49 HCs. AD, HC and MCI criteria used to recruit subjects in this study are described in our earlier work [4],[13]. Briefly, cognitive assessment was performed according to routine procedures utilized at the Fundació ACE Memory Clinic (Barcelona, Spain), as described elsewhere [14]. MCI subjects fulfilled the Petersen’s diagnostic criteria [15], including subjective memory complaints, normal general cognition, preserved performance in activities of daily living, absence of dementia and a measurable impairment in memory function, with or without deficit in other cognitive domains [16]. All MCI subjects had a Clinical Dementia Rating (CDR) of 0.5. On the basis of the Cut-off scores of a Brief Neuropsychological Battery (NBACE, a subtest of the Wechsler Memory Scale III) [14], impaired delayed verbal recall for which recognition testing does not improve performance classifies patients with amnesic MCI as having an “encoding/storage” pattern of memory loss. The diagnosis of AD was made according to National Institute of Neurological and Communicative Disorders and Stroke and the Alzheimer’s Disease and Related Disorders Association [17],[18] criteria, where AD is defined as a CDR of 1 point or more and a Mini Mental State Examination (MMSE) score below 24. HCs were cognitively normal when evaluated at the Fundació ACE Memory Clinic, had MMSE scores of at least 26 (taking into consideration the MMSE cutoff of <25 in the Spanish population [19]) and had a normal neuroimaging MRI profile.

Subject demographic characteristics are listed in Table 1. Written informed consent was obtained from each participant or, in several AD patients, the closest relative. The study protocols were reviewed and approved by the ethics committee of the Hospital Clinic i Provincial (Barcelona, Spain).

**Table 1:** **Study demographics of the AB128 study subjects**^**a**^

	**HC**	**MCI**	**AD**
Number of subjects	49	33	14
Age (yr)	56.2 (5.6)	74.6 (6.4)	79.5 (5.3)
Education (% >8 yr)	94	33	64
Gender (% males)	26.5	27.3	28.6
ApoE (% ε4 allele)	64.3	57.6	64.3
Creatinine (mg/dl)	0.77 (0.11)	0.83 (0.2)	0.89 (0.27)
DA Aβ(1–40) (pg/ml)	38.9 (10.5)	58.1 (16.3)	50.3 (18.7)
TP Aβ(1–40) (pg/ml)	83.3 (18.0)	93.0 (16.3)	93.8 (19.5)
CP Aβ(1–40) (pg/ml)	58.0 (10.3)	55.0 (14.1)	64.4 (10.7)
DA Aβ(1–42) (pg/ml)	13.5 (14.8)	12.1 (15.0)	12.4 (9.4)
TP Aβ(1–42) (pg/ml)	52.2 (33.5)	49.7 (40.9)	55.8 (30.4)
CP Aβ(1–42) (pg/ml)	164.6 (77.0)	154.6 (71.1)	163.3 (54.5)

^a^AD: Alzheimer’s disease; ApoE: Apolipoprotein E; Aβ: Amyloid-β; CP: Cellular pellet; DA: Directly accessible; HC: Healthy control; MCI: Mild cognitive impairment; RP: Recovered from plasma; TP: Total in plasma. Mean value is the number reported and the standard deviation is in brackets, as applicable.

### 2.2 Blood sampling and biochemical determinations

Blood samples from each participant were drawn on the morning after an overnight fast and were collected in polypropylene vials with ethylenediaminetetraacetic acid and a protease inhibitor cocktail (Complete Mini; Roche, Madrid, Spain). The samples were immediately cooled to 4°C until processing, which occurred within 24 hours after collection. Blood samples were centrifuged, and both the plasma and the cellular pellet (CP) were divided into aliquots and stored in polypropylene tubes at −80°C until analyzed. The material was not thawed or refrozen at any time. All samples were analyzed in triplicate in the same run for each of the three blood fractions using two specific sandwich ELISA kits, ABtest 40 and ABtest 42 (Araclon Biotech, Zaragoza, Spain), as described elsewhere [4]. Before analysis, plasma and blood cell samples were pretreated using dilution in a formulated saline buffer with 1% blocking polymer according to the supplier’s instructions. We carried out three counts for both the Aβ40 and Aβ42 peptides in each blood sample. One count was performed using the undiluted plasma sample, another using the plasma sample diluted 1:3 with the aforementioned formulated buffer and a third using the CP that remained after plasma collection. The peptide amount in the undiluted plasma sample corresponded to the directly accessible (DA) peptide. The 1:3 dilution of the plasma was chosen because it provided the maximum peptide recovery from the sample (that is, the total in plasma (TP)). Thus, this count included the DA peptide and the peptide that was recovered from the plasma matrix. Additionally, the peptide associated with the CP was measured in a 1:5 dilution of the pellet that remained after plasma collection. The sum of these three amounts is described as the total Aβ pool in blood for either Aβ(1–40) or Aβ(1–42).

### 2.3 Brain imaging and magnetic resonance imaging analysis

All MRI scans were performed with a 1.5-T MRI scanner (Magnetom Symphony; Siemens Medical Solutions, Erlangen, Germany) at the Department of Diagnostic Imaging, Corachan Clinic, Barcelona. The protocol for the acquisition of the MRI data was identical for all patients and consisted of three-dimensional T1-weighted sagittal Magnetization-prepared rapid acquisition with gradient echo, two-dimensional (2D) axial T2-weighted turbo spin echo, 2D axial fluid-attenuated inversion recovery, 2D axial T2*-weighted gradient echo and 2D axial diffusion-weighted imaging. Brain images were also visually inspected by experienced clinicians who were blinded to the participants’ demographic, anthropometric and clinical data.

Cortical reconstruction and volumetric segmentation were performed with the Freesurfer image analysis suite, which is documented and freely available for download online. The technical details of these procedures are also described in prior publications (see [20],[21] and references therein).

Briefly, this processing method includes motion correction and averaging of multiple volumetric T1-weighted images (when more than one image is available), removal of nonbrain tissue performed by using a hybrid watershed/surface deformation procedure, an automated Talairach transformation, segmentation of the subcortical white matter and deep gray matter volumetric structures (including hippocampus, amygdala, caudate, putamen and ventricles), intensity normalization, tessellation of the gray-matter–white-matter boundary, automated topology correction and surface deformation following intensity gradients to optimally place the gray–white and gray–cerebrospinal fluid borders at the location where the greatest shift in intensity defines the transition to the other tissue class.

Once the cortical models are complete, a number of deformable procedures can be performed for further data processing and analysis, including surface inflation, registration to a spherical atlas by utilizing individual cortical folding patterns to match cortical geometry across subjects, parcellation of the cerebral cortex into units based on gyral and sulcal structures and creation of a variety of surface based data including maps of curvature and sulcal depth. With this method, both intensity and continuity information from the entire three-dimensional MR volume in segmentation and deformation procedures to produce representations of cortical thickness, which are calculated as the closest distance from the gray-matter–white-matter boundary to the gray-matter–CSF boundary at each vertex on the tessellated surface. The maps are created using spatial intensity gradients across tissue classes and are therefore not reliant on absolute signal intensity only. The maps produced are not restricted to the voxel resolution of the original data; thus, they are capable of detecting submillimetric differences between groups. It should be noted that the procedures described here for the measurement of cortical thickness have been validated against histological analysis [22] and manual measurements [23],[24]. Freesurfer morphometric procedures have been proven to show good test–retest reliability across scanner manufacturers and across field strengths [20],[21].

### 2.4 Alzheimer’s Disease Neuroimaging Initiative data

For later comparison of results obtained in the AB128 project, the ADNI repository was explored for subjects with full data for MRI, Aβ plasma, age, gender and apolipoprotein E (ApoE), education and creatinine levels. Following these criteria, the data of 630 subjects were downloaded from the ADNI repository [1]. The demographic characteristics of these subjects are shown in Table 2. Other details of the ADNI cohort can be found online.

**Table 2:** **Alzheimer’s Disease Neuroimaging Initiative subject demographics**^**a**^

	**HC**	**MCI**	**AD**
Number of subjects	185	307	138
Age (yr)	76.0 (5.1)	74.8 (7.4)	75.3 (7.5)
Education (yr)	16.2 (2.8)	15.8 (3.0)	14.6 (3.2)
Gender (% males)	53.0	63.2	50.7
ApoE (% ε4 allele)	26.5	53.1	65.9
Creatinine (g/L)	114.0 (73.5)	114.3 (69.5)	105.6 (62.6)
Aβ(1–40) (pg/ml)	151.4 (49.7)	152.0 (55.7)	152.2 (40.1)
Aβ(1–42) (pg/ml)	37.8 (12.2)	36.4 (11.8)	36.4 (10.0)

^a^AD: Alzheimer’s disease; ApoE: Apolipoprotein E; Aβ: Amyloid-β; HC: Healthy control; MCI: Mild cognitive impairment. Mean value is the number reported and the standard deviation is in brackets, as applicable.

Aβ(1–40) plasma concentrations and Aβ(1–42) plasma levels of the ADNI project subjects were measured using module A of the INNO-BIA plasma Aβ forms immunoassay kit (Innogenetics, Ghent, Belgium; for research use–only reagents) on the Luminex 100 immunoassay platform and IS v.2.3 software (Luminex, Austin, TX, USA) with a fully automated sample preparation approach [25],[26]. MRI scan processing was performed with the Freesurfer image analysis software suite as explained above.

### 2.5 Statistical analysis

All the statistical procedures were carried out using R software [27]. Because of the shape of the empirical amyloid data distributions, the amyloid fractions were assumed following a lognormal distribution. Logarithms of Aβ concentrations were used in calculations. Because logarithms must be used only for dimensionless quantities, the Aβ fractions were transformed into dimensionless numbers by dividing them by a constant. Thus, for any fraction, a new quantity Aβ^ln^ was calculated as $A\beta^{\text{ln}}=\ln\left(\frac{A\beta_{i}}{\langle \mathrm{A\beta}\rangle }\right)$, where *i* runs over individuals and 〈*Aβ*〉 is the median value for the fraction. This new quantity characterized the amyloid fraction distributions in plasma for the present study.

Pearson partial correlations were calculated between the MRI measurements for 45 ROIs given in the Freesurfer subcortical atlas and different Aβ^ln^ values with age, gender and ApoE, education and creatinine levels [28]-[30] as covariates. The same procedure was applied to ADNI data. Partial correlations were calculated with ApoE, age, gender and education and creatinine levels as covariates.

Pearson partial correlations adjusted for the covariates age, gender and ApoE, education and creatinine levels were also calculated between Aβ^ln^ values and cortical volumes and cortical thickness average for 68 ROIs in the Freesurfer cortical atlas. There was no correction for multiple comparisons in any case.

The whole procedure was repeated for both data sets, AB128 and ADNI, taking age, gender and ApoE, education and creatinine levels and phenotype as covariates. Partial correlations were calculated between the 45 Freesurfer subcortical atlas ROI volumes and Aβ^ln^ values and also between the 68 Freesurfer cortical atlas ROI volumes and thickness average values and Aβ^ln^ values. No correction for multiple comparisons was done.

An additional analysis was performed to determine the association between Aβ^ln^ CP Aβ(1–40) values and more correlated brain ROIs was conducted. The subjects were divided into phenotype groups (AD, MCI and HC), and each group was analyzed separately. Linear regressions were performed between Aβ^ln^ CP Aβ(1–40) and left hippocampal volume in each case.

Finally, Pearson partial correlations adjusted for the covariates age, gender and ApoE, education and creatinine levels were also calculated between Aβ^ln^ values for each amyloid fraction and MMSE score.

## 3 Results

Partial Pearson correlations were calculated for 45 ROIs contained in the Freesurfer subcortical atlas, and Aβ^ln^ compartments with age, gender and ApoE, education and creatinine levels were taken as covariates (see Additional file 1: Table S1a). The left hippocampal volume was the top brain ROI for Aβ^ln^ CP Aβ(1–40) fraction (pcor = −0.37, *P* =0.0007), as shown in Table 3. Furthermore, the only Aβ^ln^ fraction that remained significant when associated with left hippocampal volume was again the Aβ^ln^ CP Aβ(1–40) fraction.

**Table 3:** **Hippocampal volume partial correlations with and without including diagnostic category**^**a**^

	**Not including diagnostic category**		**Including diagnostic category**	
	**Partial correlation**	***P*-value**	**Partial correlation**	***P*-value**
Aβ^ln^ DA Aβ(1–40)	0.05	0.64	−0.01	0.92
Aβ^ln^ DA Aβ(1–42)	−0.08	047	−0.15	0.20
Aβ^ln^ TP Aβ(1–40)	0.03	0.78	0.01	0.92
Aβ^ln^ TP Aβ(1–42)	−0.01	0.37	−0.14	0.22
**Aβ**^**ln**^CP Aβ(1–40)*	**−0.37**	**0.0007**	**−0.35**	**0.002**
Aβ^ln^ CP Aβ(1–42)	−0.16	0.17	−0.2	0.07
Total Aβ1	−0.18	0.11	−0.24	0.03
ADNI Aβ^ln^ Aβ(1–40)	0.08	0.05	0.1	0.01
ADNI Aβ^ln^ Aβ(1–42)	0.08	0.04	0.09	0.02

^a^ADNI: Alzheimer’s Disease Neuroimaging Initiative; Aβ: Amyloid-β; CP: Cellular pellet; DA: Directly accessible. The data are derived from the AB128 and ADNI data sets and represent partial correlations of Aβ blood levels with left hippocampal volume adjusted for the covariates age, gender, ApoE, education, creatinine levels and phenotype. The left columns show the correlations and *P*-values if phenotype is excluded as covariate. ADNI Aβ^ln^ Aβ(1–40) and Aβ^ln^ Aβ(1–42) values are placed underneath their comparative quantities. *Bold text highlights the correlation of the Aβ^ln^ CP Aβ(1–40) fraction in the AB128 study.

The same procedure was repeated between the plasma Aβ^ln^ values and 68 ROI volumes from the Freesurfer cortical atlas and thickness average segmented in Freesurfer (Additional file 1: Tables S1b and S1c). Here the left hemisphere entorhinal volume (pcor = −0.3, *P* = 0.008) and left hemisphere entorhinal thickness average (pcor = −0.2, *P* = 0.06) emerged as the most significant cortical measurements for Aβ^ln^ CP Aβ(1–40).

Pearson correlations were repeated, but this time also including phenotype as a covariate (see Additional file 2: Table S2). Again, the left hippocampus was the top brain ROI for Aβ^ln^ CP Aβ(1–40) fraction (pcor = −0.35, *P* =0.002) for subcortical volumes, as shown in Table 3. Left hemisphere entorhinal volume (pcor = −0.3, *P* =0.008) was the most significant of the cortical volumes (pcor = −0.27, *P* =0.02) and thickness average (pcor = −0.2, *P* =0.1) for Aβ^ln^ CP Aβ(1–40).

Unfortunately, the top associations detected were related to cell-bound Aβ fractions, which are not measured in the conventional assays available. A similar analysis using the ADNI data set was performed, although Aβ levels measured in that series were restricted to a single plasma measurement (equivalent to TP fraction in our assay) adjusted for the covariates age, gender, ApoE, education and creatinine levels, and no association was found. In addition, no association was obtained when the analysis was adjusted for the covariates age, gender, ApoE, education, creatinine levels and diagnostic category. These results are fully compatible with our findings in TP. The partial correlations between Aβ^ln^ values and left hippocampal volume adjusted for the covariates age, gender and ApoE, education and creatinine levels are shown in Table 3. The partial correlations when phenotype was added as a covariate are also shown in Table 3.

**Figure 1:**
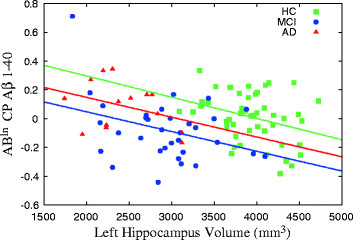
**Hippocampal volume versus amyloid regression.** Graph shows results of linear regression analysis of the left hippocampal volume and amyloid-β (Aβ) levels of Aβ^ln^ cellular pellet (CP) Aβ(1–40) in the healthy control (HC), minor cognitive impairment (MCI) and Alzheimer’s disease (AD) groups.

Stratification analyses by phenotypic groups suggested that each diagnostic group had a similar correlation with left hippocampal volume in terms of effect size and significance. In other words, the observed correlations cannot be attributed to any specific cognitive subgroup, as shown in Figure 1. Pearson partial correlation analysis of Aβ^ln^ values and MMSE scores showed no correlation between both magnitudes.

## 4 Discussion

The major finding of this study is that cell-bound Aβ was correlated with left hippocampal volume, a major area of AD pathology. Therefore, even when cell-bound Aβ levels do not distinguish HC, MCI and AD satisfactorily [4], they are related to their physiological counterpart, hippocampal damage. Diluted and undiluted plasma Aβ levels did not significantly correlate with hippocampal volume. This result is important because most studies of Aβ in blood have included analysis of only plasma levels, and the most important Aβ carriers in the blood, which are the cell membranes, have been systematically ignored [12]. We used Aβ plasma and MRI measurements taken from the ADNI project and observed a relationship similar to the one in our data. The plasma Aβ fractions have proven to be unrelated to any specific brain region in the ADNI or AB128 data sets. This fact points to a different behavior, biochemically, for the Aβ(1–40) and Aβ(1–42) biomarkers measured with the traditional kit as well as the CP Aβ(1–40) fraction measured in this study.

On the other hand, the relationship we found seems to be independent of subject phenotype. Indeed, the similarity between the correlations with and without phenotype as covariates points out that the relationship to hippocampal volume is purely physiological. The same conclusion can be extrapolated from the slopes of linear regressions between left hippocampal volume and Aβ^ln^ CP Aβ(1–40) level shown in Figure 1. Notice that the slopes represent approximately the same value for each group. Additional confirmation is provided by the fact that no correlation was found between MMSE score and Aβ^ln^ CP Aβ(1–40) level.

The unexpected association between CP Aβ(1–40) level and left hippocampal volume was examined exhaustively and, after a statistical analysis, proved to be consistent enough to be reported. This newly established relationship deserves further research. Even when replications were needed, the *ad hoc* analysis showed that blood CP Aβ(1–40) level could be used as a suggestive proxy for hippocampal volume and thus could be a useful screen for AD, even if it must be used with other biomarkers. Further studies are necessary to validate this working hypothesis.

The major limitation of this study is the sample size. The study in fact involved a modest number of subjects. Only independent replications will be able to help clarify whether the observed associations and statistical significance are related to true findings or to random statistical oscillations. If we can finally confirm this observation, our findings may open new avenues to developing an AD biomarker related to the intrinsic properties of Aβ bound to blood cells. This finding might also have several physiological and pathological implications beyond the scope of this study. Another important limitation of the study is that no correction for multiple comparisons was done. Therefore, further studies are necessary to clarify the effect and function of Aβ peptides bound to different blood cells.

## 5 Conclusions

The cell-bound Aβ(1–40) fraction was found to correlate with volume in the left hippocampus, a major site of AD pathology. However, other plasma Aβ fractions did not correlate with hippocampal volume. This suggests a different behavior, biochemically, for the traditionally measured Aβ(1–40) and Aβ(1–42) biomarkers and the CP Aβ(1–40) fraction. This newly established relationship deserves further research. Blood CP Aβ(1–40) may prove to be a useful screening test for AD, even as part of a composite biomarker.

## Abbreviations

AD: Alzheimer’s disease

ADNI: Alzheimer’s Disease Neuroimaging Initiative

ApoE: Apolipoprotein E

Aβ: Amyloid-β

CDR: Clinical Dementia Rating

CP: Cellular pellet

CSF: Cerebrospinal fluid

DA: Directly accessible

ELISA: Enzyme-linked immunosorbent assay

HC: Healthy control

MCI: Mild cognitive impairment

MMSE: Mini Mental State Examination

MRI: Magnetic resonance imaging

ROI: Region of interest

RP: Recovered from plasma

TP: Total in plasma

## Competing interests

AR is a shareholder of Neopharm Obesity and Oxigene. PP, ISJ, VPG and MS are employees of Araclon Biotech Ltd. MS and ISJ are shareholders of Araclon Biotech Ltd. MBo is a consultant to Novartis and Esteve Pharmaceuticals; she is supported in part by FIS/EC 11-358 and FIS/P 10-00945 funds from Ministerio de Sanidad, Servicios Sociales e Igualdad, Spain (AATM/390-6-2009), Generalitat de Catalunya (Catalan government); and she is a member of Advisory Boards of Grifols, Lilly, Elan, Nutricia, Genentech and Roche. OSG, SV, IH, AL, MBu, MI, MAT, JG and LT have nothing to disclose. This work was funded by Araclon Biotech And Fundació ACE Memory Clinic and was also supported by the Spanish Ministry of Health through Instituto de Salud Carlos III (Madrid) (FISS PI10/00954) and by Agència d’Avaluació de Tecnologia i Recerca Mèdiques, Departament de Salut de la Generalitat de Catalunya (grant 390). Fundació ACE Memory Clinic is a CIBERNED-associated site.

## Authors’ contributions

MS, MBo, LT, ISJ, PP, AR and OSG conceived of and designed the experiments. PP, ISJ, VPG, MAT, JG, AL, MBu, MI and IH performed the experiments. OS, SV, AR, PP, MBo, LT and MS analyzed the data. PP, VPG, SV, OS and AR contributed reagents/materials/analysis tools. OS, AR, PP, MS, MBo and LT wrote the manuscript. All authors read and approved the final manuscript.

## Additional files

## Supplementary Material

Additional file 1: Table S1. Excel spreadsheet with partial correlations adjusted for the covariates age, ApoE, gender, education and creatinine levels. (a) Partial Pearson correlations between amyloid-β fractions and volume Freesurfer subcortical ROIs. (b) Partial Pearson correlations between amyloid-β fractions and volume of Freesurfer cortical ROIs. (c) Partial Pearson correlations between amyloid-β fractions and cortical thickness of Freesurfer cortical ROIs. Click here for file

Additional file 2: Table S2. Excel spreadsheet with partial correlations adjusted for the covariates age, ApoE, gender, education, creatinine levels and phenotype. (a) Partial Pearson correlations between amyloid-β fractions and volume Freesurfer subcortical ROIs. (b) Partial Pearson correlations between amyloid-β fractions and volume of Freesurfer cortical ROIs. (c) Partial Pearson correlations between amyloid-β fractions and cortical thickness of Freesurfer cortical ROIs. Click here for file
